# Response of forest *Turtur* doves to conspecific and congeneric songs in sympatry and allopatry

**DOI:** 10.1038/s41598-023-43035-8

**Published:** 2023-09-24

**Authors:** Małgorzata Niśkiewicz, Paweł Szymański, Michał Budka, Tomasz S. Osiejuk

**Affiliations:** Department of Behavioural Ecology, Institute of Environmental Biology, Faculty of Biology, Adam Mickiewicz University, Uniwersytetu Poznańskiego 6, 61-614 Poznań, Poland

**Keywords:** Animal behaviour, Behavioural ecology, Evolutionary ecology, Tropical ecology

## Abstract

Birds have a diverse acoustic communication system, and the ability to recognise their own species’ song from a distance facilitates complex behaviours related to mate attraction and rival deterrence. However, certain species, including doves, do not learn songs and their vocal repertoires are much simpler than those of better-studied songbirds. In these so-called non-learning birds, relatively little is known about the role that bird song plays in intra- and interspecific interactions, and how such behaviours might be acquired (inherited or learned from experience). To investigate this question, we focused on two species of African wood doves whose long-range songs are used in a territorial context. Specifically, we examined the responses of sympatric and allopatric populations of male blue-headed wood-doves (*Turtur brehmeri*) and tambourine doves (*Turtur tympanistria*) to different types of simulated territorial intrusions, i.e. playback of conspecific, congeneric, and control songs. We aimed to assess (i) whether these species, which have similar songs, respond only to their own species' song or exhibit interspecific territoriality, and (ii) if the response pattern is affected by the presence or absence of congeners in the general area. We found that both species responded strongly to playback of their own species in both sympatric and allopatric populations. In allopatry, though, male tambourine doves misdirected their response and also approached the playback of congeneric songs. Our results indicate that, in areas where the studied *Turtur* doves live in sympatry, they do not exhibit consistent interspecific territoriality. However, we cannot exclude the possibility that the smaller tambourine dove avoids its larger congener during the process of territory establishment. The difference in tambourine doves’ response toward the song of present (sympatric) or absent (allopatric) congeners suggests that the ability to discriminate between songs of similarly singing potential competitors is acquired through earlier interactions and learning. This plasticity in response supports the misdirected aggression hypothesis, which argues that interspecific territorialism emerges as a maladaptive by-product of signal similarity. However, on an evolutionary timescale, such an ability could be considered an adaptive cognitive tool useful for resolving competing interests with congeners.

## Introduction

Acoustic communication is widespread in many animal taxa, but the evolution of complex sound signals has occurred in only a few groups. One such group is birds, whose songs exhibit an extraordinary degree of diversity and sophistication^1,2^. Apart from some basic pre-adaptations for vocal communication found in the ancestors of modern birds (e.g., breathing apparatus), the main factor that led to the evolution of elaborate songs was sexual selection. To wit, the most important functions of bird song are attracting a breeding partner and defending territory or other resources^2^. These two functions are not mutually exclusive and were historically assigned to males, as males were thought to be the dominant singing sex^3^. To fulfil these functions, songs must be species-specific as well as sufficiently individually unique to enable discrimination or recognition of individuals or classes of individuals (e.g., stranger vs neighbour)^2,4^. This two-level specificity of the song is then interpreted by the receiver for use in decision making, even in the absence of visual information^5^.

In species where only the male sings, the meaning of a song could be different from the perspectives of male and female receivers. When a female recognises the song of her own species, she can, in the next step, evaluate the quality (or other features like dialect origin) of the sender and decide whether to reproduce with him^6,7^. A male receiver, on the other hand, is usually focused on extracting information about the threat level expressed by the signal. For example, when territory boundaries are established, the song of a neighbour singing from his territory border is usually treated as less dangerous than the song of a stranger^8^. The evaluation of a potential rival's song enables the territory owner to make an appropriate decision about interacting with that rival^9,10^. Responding too aggressively may lead to a risk of injury or death in the worst case or wasted energy in the best case^10,11^. For the territory owner and potential mate, a long-distance signal carrying a message about species and individual identity is usually crucial and a prerequisite for territorial behaviour^2^.

### Interspecific territoriality

Territoriality is widespread among animals^12–15^. Within a species, individuals prefer similar habitats and must compete for limited resources, but the exact pattern and level of aggressiveness exhibited during competition depend on their life histories (e.g., sedentary vs migratory lifestyle, breeding system)^16,17^. Initially, between-species interactions were assumed to be rare, costly, and the result of ancestral signal similarity. Surprisingly, recent comparative studies have revealed that interspecific territoriality is common in birds^18,19^. To explain the evolution of interspecies territorialism, four basic hypotheses have been proposed (reviewed in Cowen et al.^20^). The first is the resource competition hypothesis, which assumes that resources are partitioned spatially between dominant individuals of competing species^21–23^. The second hypothesis, asymmetric competition, is also based on strong resource rivalry but assumes that only dominant males of the dominant species succeed in interspecific territoriality^24^. The reproductive interference hypothesis explains the existence of territorial aggression between species as the result of competition not for the same resources but for a partner, due to incomplete reproductive isolation and the possibility of hybridisation^25,26^. The last hypothesis, misdirected aggression, posits that interspecies territorialism is maladaptive and a by-product of intraspecies territorialism^15,27^.

These hypotheses reflect field observations and are not mutually exclusive. To shed light on this phenomenon, then, researchers must search for evolutionary scenarios in which these different options are possible, rather than try to eliminate erroneous hypotheses. Interspecific territoriality is more likely if species have somewhat similar songs and can physically interact^20^. Hence, the first step for understanding the process of such territoriality is examining if a species detects the signal of a congeneric and recognises it as worthy of a response. The hypotheses presented above provide some background for when, why, and how this might happen. As predominantly territorial animals, birds are particularly interesting models for studying interspecific territoriality because different groups of species are diametrically opposed to each other in the way acoustic signals are transmitted between generations (inherited vs learned). In addition, territorial defence in birds involves processing signals heard from a distance, and at least the initial part of the response is evoked solely by the sound signal^2^.

### Aims of the study

One interesting scenario for comparing potential interactions between species is when closely related species exploit similar resources and have similar signals. In such a case, interspecific interactions may be the result of competition, leading to interspecific territoriality and an intentional response to the other species (including avoidance of a 'stronger' competitor). However, interactions could also, at least potentially, be due to mistakes (similar signals). In addition, the character of such interactions might depend on whether the studied pair of species naturally occur in the same location (sympatry) or not (allopatry).

**Figure 1:**
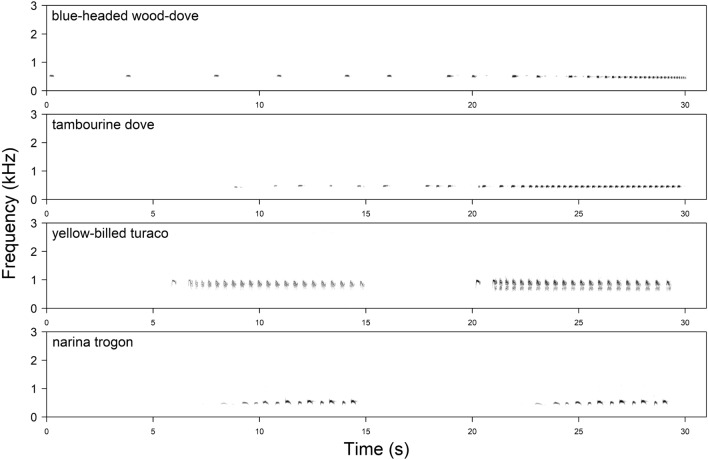
Spectrograms of the songs of focal bird species: blue-headed wood-dove (*Turtur brehmeri*) and tambourine dove (*Turtur tympanistria*), and species used as a control: yellow-billed touraco (*Tauraco macrorhynchus*) and narina trogon (*Apaloderma narina*).

In this study, we experimentally tested the response of two non-learning, territorial bird species to conspecific and congeneric songs in both the sympatric and allopatric ranges. The main objectives were to see whether and how the studied species respond to each other's songs and how this response might change in areas in which the rival is not naturally found. Our models were the blue-headed wood-dove, *Turtur brehmeri* (hereafter *brehmeri*), and the tambourine dove, *T. tympanistria* (hereafter *tympanistria*). Genus *Turtur* contains a total of five species living in varied habitats of sub-Saharan Africa, and all have very similar songs that are used for long-distance communication. The songs consist of simple, low-frequency notes organised with generally similar syntax (songs have two parts differing in the duration of syllables, pauses, and peak frequency) and are quite different in comparison to other doves (Fig. 1). The model species both inhabit forests, but *brehmeri* has a range limited to central and western Africa, while *tympanistria* can be found in varied, forested habitats in almost all of sub-Saharan Africa^28,29^.

In the area of sympatric occurrence, we tested both dove species. We expected birds to demonstrate a strong response to their own species’ song. If the two species indeed compete in these habitats, the smaller species should avoid the larger intruder. For this reason, we predicted that *tympanistria* males would not respond to the congener's song or might even exhibit extreme avoidance behaviours (like flying away and ceasing to sing). Instead, the larger *brehmeri* should attempt to chase away its smaller competitor. Thus, we also expected that *brehmeri* males might respond to the *tympanistria* song, but not necessarily as strongly as to that of conspecific rivals. If the species do not exhibit interspecific territoriality, however, we would not expect to observe any response to the songs of congeners.

For our allopatric sites, we conducted experiments in a forest where only *tympanistria* occurs. Again, we expected a strong response to the conspecific song. Regarding the response to the absent congener, we investigated the possibility of two potential scenarios. Males of *tympanistria* may not respond to an unknown congener’s song if it is not recognised as a potential competitor. Such a result would suggest that recognition of species-specific songs is inherited, and the relatively small differences between *tympanistria* and *brehmeri* songs facilitate efficient recognition even in the absence of any earlier experience with the congeneric species. However, if the allopatric *tympanistria* males respond to the song of a congener, it would support the misdirected aggression hypothesis, i.e., it is likely to be a case of mistaken identity due to the general similarity of both species' songs. It would also imply that any kind of response found between *tympanistria* and *brehmeri* in sympatry must result from earlier interactions between individuals of both species.

## Methods

### Study sites and species

The study was conducted in two sites in sub-Saharan Africa, one where *brehmeri* and *tympanistria* occur in sympatry and one where only *tympanistria* is present. The first site was in Kakum National Park, in the coastal region of southern Ghana (5.20–5.40 N, 1.30–1.51 W, altitude 135–250 m asl). The park covers an area of ~ 210 km^2^ of tropical moist evergreen forest with fragments of periodic or permanent swamp and riverine forests. Although logging operations between 1975 and 1989 decreased areas of dense primary forest, the logged areas have regenerated secondary forests characterised by denser vegetation. Kakum NP receives an annual average rainfall of 1460 mm, with most precipitation in September–October and March–June. Temperatures vary between 20 and 30 °C^30,31^. The second site was in Kibale National Park in Uganda (0.13–0.41 N, 30.19–30.32 E, altitude 1100–1600 m asl), a moist, evergreen, medium-altitude forest with a mosaic of primary and secondary forest, swamp, grassland, and thicket. Temperatures are very stable over the course of the year (daily fluctuation 15–27 °C) and the precipitation (annually around 760 mm) has a bimodal pattern, with more rainfall in March–April and September–November^32^. There appears to be no detectable bird seasonality in this area^33^.

In this study, we focused on two *Turtur* species that prefer strictly forest-like habitats. The more ubiquitous *tympanistria* is typically found in any type of forest or forest-like habitat, such as lowland and montane forests (even up to 3000 m asl), secondary forests, gardens, plantations, or even mangroves^29^. The range of *brehmeri* is restricted to western Africa and lowland primary forests up to 750 m asl only. Occasionally it is also found in old secondary forests, but generally, it avoids heavily disturbed forests^28^. With a body mass of 98.5–129.5 g (*N* = 10), *brehmeri* are significantly (t_2,50_ = 19.6, *P* < 0.0001) larger than *tympanistria* (51.5–81 g; *N* = 42; own measurements in the study area). From our own observations and the literature, we found no evidence of hybridisation between these two species^34^. Furthermore, a STRUCTURE analysis performed on 7000 SNP markers (obtained by sequencing RADseq libraries) did not detect any signal of admixture between *brehmeri* and *tympanistria* (own unpublished data based on 6 *brehmeri* and 26 *tympanistria* individuals). We did not observe any direct, physical interaction between males of these species in the sympatric area, although both species were heard singing relatively close to each other. However, the above observations do not exclude the possibility of interspecific territoriality completely.

### Playback stimuli

We prepared three types of playback: (i) conspecific (*brehmeri* and *tympanistria* for *brehmeri* and *tympanistria*, respectively), (ii) congeneric (*brehmeri* for *tympanistria* and *tympanistria* for *brehmeri*), and (iii) a control (yellow-billed tauraco, *Tauraco macrorhynchus*, in Kakum NP and narina trogon, *Apaloderma narina*, in Kibale NP). The control species were chosen because their territorial calls have similar spectra to the territorial songs of doves. Both species are common in the studied areas but are not perceived as a threat by doves and do not interact with them. To create playback stimuli of doves, we selected recordings of territorial songs of local non-neighbouring males with a high signal-to-noise ratio. Each song sample was filtered (high-pass, 0.1 kHz; low-pass, 1.5 kHz). Each playback stimulus was created from a sample belonging to a different individual to avoid pseudo-replication. Dove playback consisted of a single song sample repeated 10 times (two songs per minute), which is the typical calling rate of both *brehmeri* and *tympanistria*. Comparable song durations and pacing were also maintained for the control treatments. The amplitude of each playback was set to 82 ± 2 dB SPL at a 1-m distance from the speaker, measured with a CHY 650 digital sound level meter (CHY Firemate Co., Ningbo, China). All playback stimuli were created with Raven Pro 1.6 (Cornell Lab of Ornithology, Ithaca, USA) and Avisoft SASLab Pro 5.2 (Raimund Specht, Berlin).

### Playback experiment design and procedures

#### General procedures of experiments I–III

Each male was tested three times (conspecific, congeneric, and control species treatments) in a counterbalanced order. Experiments were only conducted when a male was observed and heard singing before the playback. In each trial with a given male, the speaker was placed in a slightly different location to avoid the habituation of the focal bird, but always within the same distance (~ 50 m) of the place from which the bird was singing before playback and at the same height above the ground, ca. 2 m. To broadcast the playback stimuli, we used either a Tascam DR-40X (TEAC Europe GmbH, Wiesebaden, Germany) or Sony PCM-D100, and JBL Charge 4 speakers (Harman International Industries, Stamford, Connecticut, USA). Before starting the 5-min playback, we recorded the focal male for at least 1 min. Recorded songs were then used for checking if the responding individuals were the same as before the start of playback. The identity check was based on comparing the time and frequency distribution of the initial syllables in songs (see Fig. S1). This method was developed for *tympanistria* and allows for even 96% classification efficacy, depending on the number of compared syllables^35^. Analogous measurements allow for differentiating between males of *brehmeri* at a similar level (unpublished own data). Playback was then followed by a 5-min post-playback phase.

Each trial was observed by two people positioned on opposite sides of the speaker, ca. 20 m away from it, in locations that minimised the possibility of disturbing a bird approaching the speaker. The person recording songs had a lavalier microphone connected to the second channel of the main recorder for dictating observations. The second observer was on the opposite side of the speaker in a place convenient for observing the whole experimental scene. The birds' behavioural responses were recorded by dictating observations into the additional recorder. Recordings of vocalisations and comments on physical behaviour were later time-aligned and combined into one audio file. All distances reported (as the closest distances to the speaker) were measured with a Leica DISTO D510 laser range finder.

Our preliminary work with the study species revealed they are shy and that observing their behaviour in the forest is difficult. Therefore, we were focused a priori on those aspects of behaviour that we were able to quantify with sufficient and repeatable certainty among all experiments. In the case of the vocal response, we used the number of songs sung by males. The physical behavioural responses of males were measured as the time spent within 25 m of the speaker (s), latency to approach the speaker (s), the closest approach to the speaker (m), and the number of flights.

#### Experiments in sympatric area

Playback for Experiment I (with *brehmeri*) and Experiment II (with *tympanistria*) was performed between November 12 and December 1 in 2021 between 6:04 and 12:43 local time (sunrise started 5:52–5:58) in Kakum NP. All males were tested three times, with the three types of songs (conspecific, congeneric, and control song of yellow-billed turaco) presented in a counterbalanced order. The time of testing reflected birds' activity and depended on the weather on a particular day (in practice, single trials were performed after 12:00 on two separate occasions when we waited longer for birds after two earlier tests). Altogether, we tested 19 males of *brehmeri* and 14 males of *tympanistria*. The average time between the following treatments with the same male was 32 min for Experiment I and 39 min for Experiment II.

#### Experiment in allopatric area

Playback for Experiment III was performed between June 28 and July 6 in 2022. We tested birds during their active time in the morning, 6:58–9:47 (sunrise started between 5:59 and 6:00), and in the late afternoon, 16:44–17:48 (sunset started between 18:03 and 18:05). Each male was tested only in the morning or the evening, as their pattern of activity was strictly bimodal and short due to weather conditions. Altogether, in Kibale NP we tested 15 males of *tympanistria*. The three songs of each trial were presented in a counterbalanced order, exactly as in Experiments I-II, and differed only in the species used for the control (narina trogon). The average time between subsequent treatments with the same male was 21 min.

### Ethical note

To our knowledge, the individuals tested in the experiment reflected the population in a representative way with no potential biases resulting from social background, self-selection, habituation, or other factors as indicated in the STRANGE framework (Webster and Rutz 2020). This study was designed and performed in accordance with the ARRIVE guidelines^36^. Our experimental procedure adhered to the ASAB/ABS Guidelines for the care and use of animals (The Ethics Committee (ASAB) and the Animal Care Committee (ABS), 2019) and was approved by all responsible local bodies listed below as well as by the Polish Laboratory Animal Science Association (certificate no. 1952/2015 to TSO, conforming to Directive 2010/63/EU). Our experimental procedures were approved in Ghana by the Forestry Commission (Wildlife Division), permit no. WD/A.185/Vol.13/80, and in Uganda by Makerere University (College of Health Sciences; Makerere University Biological Field Station) and the Uganda Wildlife Authority, permit no. COD/96/05 and Research Material Transfer MTA no. 377, as well as the Uganda National Council for Science and Technology, permit no. NS256ES.

### Statistical analysis

The original response variables we measured during experiments were partly correlated with each other, but the multicollinearity was moderate (variance inflation ratio VIF between 1.02 and 3.42). Therefore, to analyse the general strength of the response to playback, we used a principal component analysis (PCA) with varimax rotation and Kaiser normalisation (IBM SPSS Statistics 28.0.1.0). We extracted principal components separately for all three experiments. All three datasets were well suited for PCA (KMO and Bartlett's tests are given in Tables 1, 2, 3); the first two extracted components explained a similar percentage of the variance (PC1: 43.77–47.93% and PC2: 25.52–30.40%) and had a similar pattern of loadings from the original variables. All the first components had heavier loadings from variables related to the approach to the speaker (Tables 1, 2, 3), while all the second components had heavier loadings from the variables related to singing. Therefore, we refer hereafter to PC1 as the ‘Approaching’ component and to PC2 as the 'Vocal response' component (Tables 1, 2, 3). Higher values of PC1 indicate more flights during the playback, a closer approach to the speaker, and more time spent in its vicinity, hence, a stronger response. Higher values of PC2 indicate that responding birds sang more songs both during and after playback.

**Table 1:** Principal component loadings for blue-headed wood-dove responses to playback in sympatry (Experiment I).

Statistics and original response variables	PC1—approaching	PC2—vocal response
Eigenvalue	2.936	1.515
% of variance	43.773	30.401
Cumulative %	43.773	74.173
Songs during playback	− 0.26	**0.88**
Songs after playback	0.01	**0.92**
Flights during playback	**0.80**	− 0.26
Flights after playback	0.66	0.26
Closest distance (m)	− **0.89**	0.22
Time < 25 m to speaker (s)	**0.83**	− 0.16

Kaiser–Meier–Olkin = 0.702, Bartlett's test of sphericity χ^2^ = 150.245, *P* < 0.001.

Values that make a substantial contribution to the overall variance are in bold.

**Table 2:** Principal component loadings for tambourine dove responses to playback in sympatry (Experiment II).

Statistics and original response variables	PC1—approaching	PC2—vocal response
Eigenvalue	2.693	1.636
% of variance	44.879	27.275
Cumulative %	44.879	72.153
Songs during playback	− 0.24	**0.85**
Songs after playback	− 0.03	**0.80**
Flights during playback	**0.95**	− 0.04
Flights after playback	0.29	0.53
Closest distance (m)	− **0.89**	0.03
Time < 25 m to speaker (s)	**0.91**	0.05

Kaiser–Meier–Olkin = 0.656, Bartlett's test of sphericity χ^2^ = 113.173, *P* < 0.001.

Values that make a substantial contribution to the overall variance are in bold.

**Table 3:** Principal component loadings for tambourine dove responses to playback in allopatry (Experiment III).

Statistics and original response variables	PC1—approaching	PC2—vocal response
Eigenvalue	2.876	1.531
% of variance	47.926	25.522
Cumulative %	47.926	73.447
Songs during playback	− 0.13	**0.91**
Songs after playback	0.30	**0.84**
Flights during playback	**0.92**	− 0.08
Flights after playback	0.53	0.13
Closest distance (m)	− **0.93**	− 0.07
Time < 25 m to speaker (s)	**0.85**	0.07

Kaiser–Meier–Olkin = 0.675, Bartlett's test of sphericity χ^2^ = 123.563, *P* < 0.001.

Values that make a substantial contribution to the overall variance are in bold.

To test for differences in the response to conspecific, congeneric, and control songs, we built generalised linear mixed-effects models (GLMM) using the 'lme4' package of R^37^ and checked model assumptions using the DHARMa package^38^. Our response variables were the measures of approaching (PC1) and vocal response (PC2) extracted separately for each experiment. We included in our models two main factors: (1) playback treatment (three levels: conspecific, congeneric, and control) and (2) playback order (three levels: first, second, or third). We included all first-order interaction terms and incorporated male identity as a random effect. For model selection, in all above analyses we adopted an information theoretic approach^39^. We ranked all possible models mentioned above according to their value of Akaike's information criterion corrected for small sample size (AICc), and obtained an averaged model by selecting the most supported ones (ΔAICc ≤ 6) after the exclusion of uninformative parameters using the MuMIn package in R^40^. Models were not over-dispersed (GLMM_Experiment I,PC1_: *P* = 0.936; GLMM_Experiment I,PC2_: *P* = 0.96; GLMM_Experiment II,PC1_: *P* = 0.92.; GLMM_Experiment II,PC2_: *P* = 1.0; GLMM_Experiment III,PC1_: *P* = 0.856; GLMM_Experiment III,PC2_: *P* = 0.88), no outliers were detected (all GLMMs: *P* = 1, except GLMM_Experiment II,PC1_: *P* = 0.285), and visual inspection of the Q-Q plots confirmed the normality of the residuals with a single exception at the marginal significance level (Kolmogorov–Smirnov test, GLMM_Experiment I,PC1_: *P* = 0.046; GLMM_Experiment I,PC2_: *P* = 0.671; GLMM_Experiment II,PC1_: *P* = 0.336; GLMM_Experiment II,PC2_: *P* = 0.964; GLMM_Experiment III,PC1_: *P* = 0.705; GLMM_Experiment III,PC2_: *P* = 0.570). We applied post-hoc contrasts to differentiate between the different levels of treatments. All *P* values reported are two-tailed.

## Results

**Figure 2:**
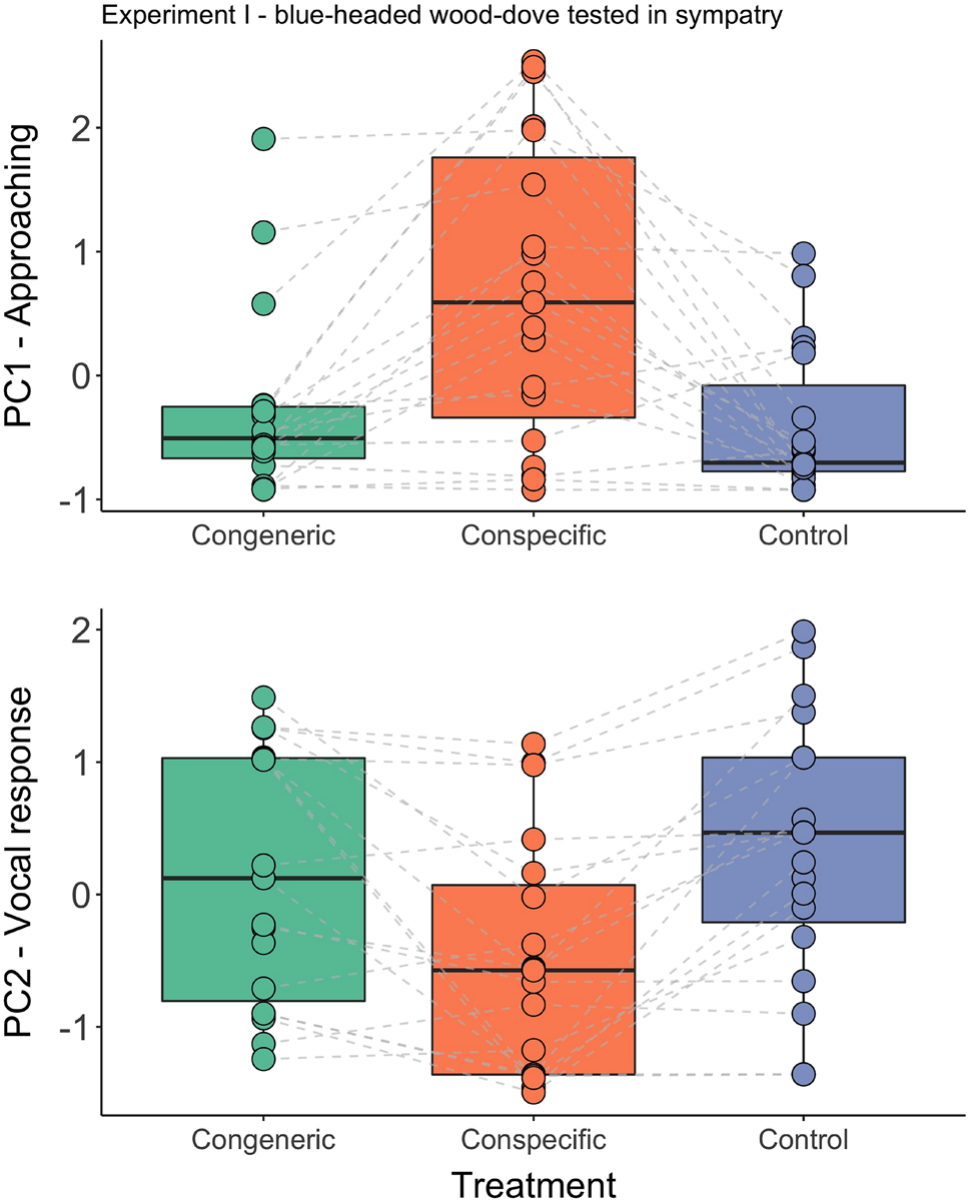
Experiment I: blue-headed wood-dove males responses to playback of conspecific, congeneric and control songs in sympatry (Ghana), measured with compound measures of response: PC1-approaching and PC2-vocal response.

**Figure 3:**
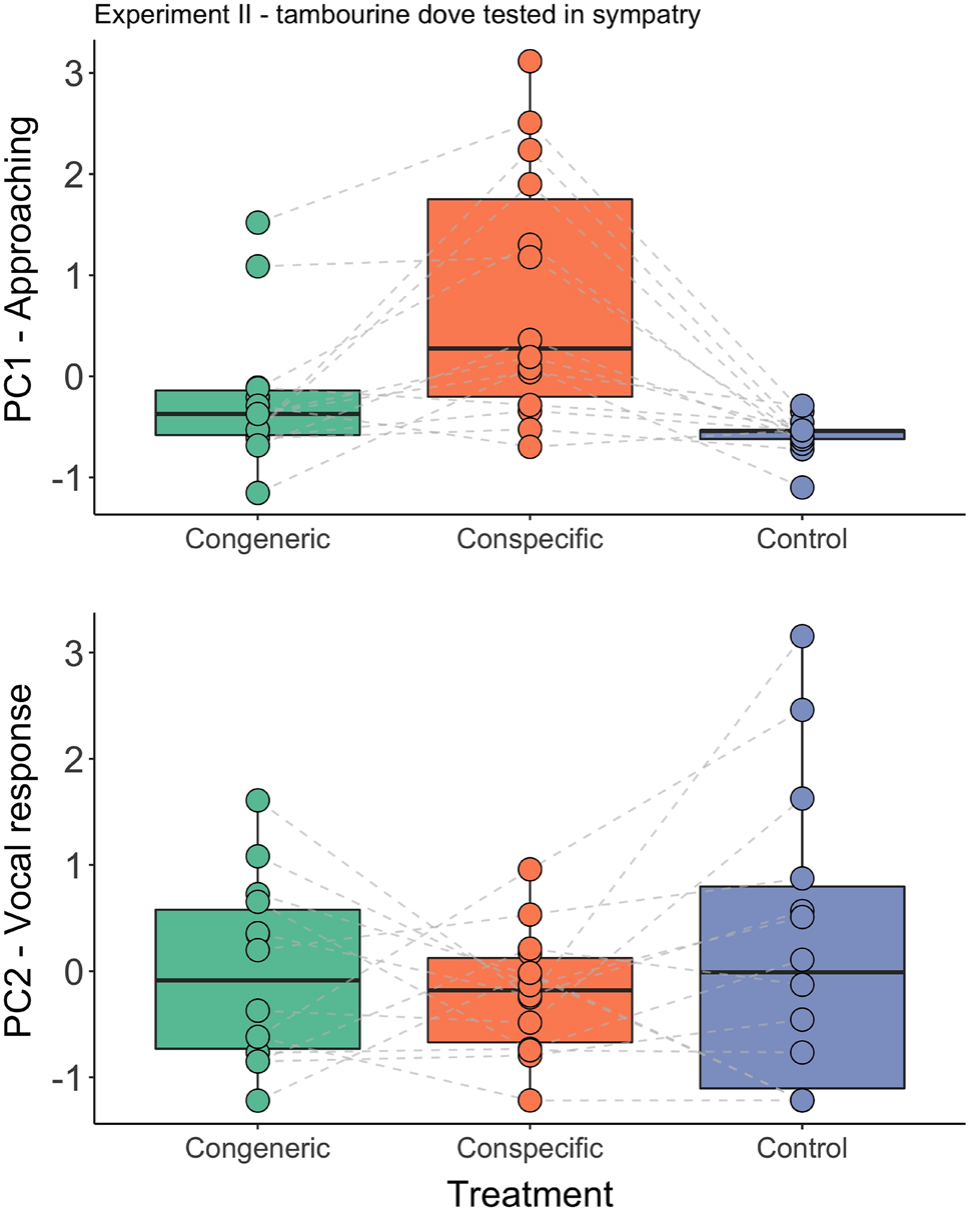
Experiment II: tambourine dove males responses to playback of conspecific, congeneric and control songs in sympatry (Ghana), measured with compound measures of response: PC1-approaching and PC2-vocal response.

**Figure 4:**
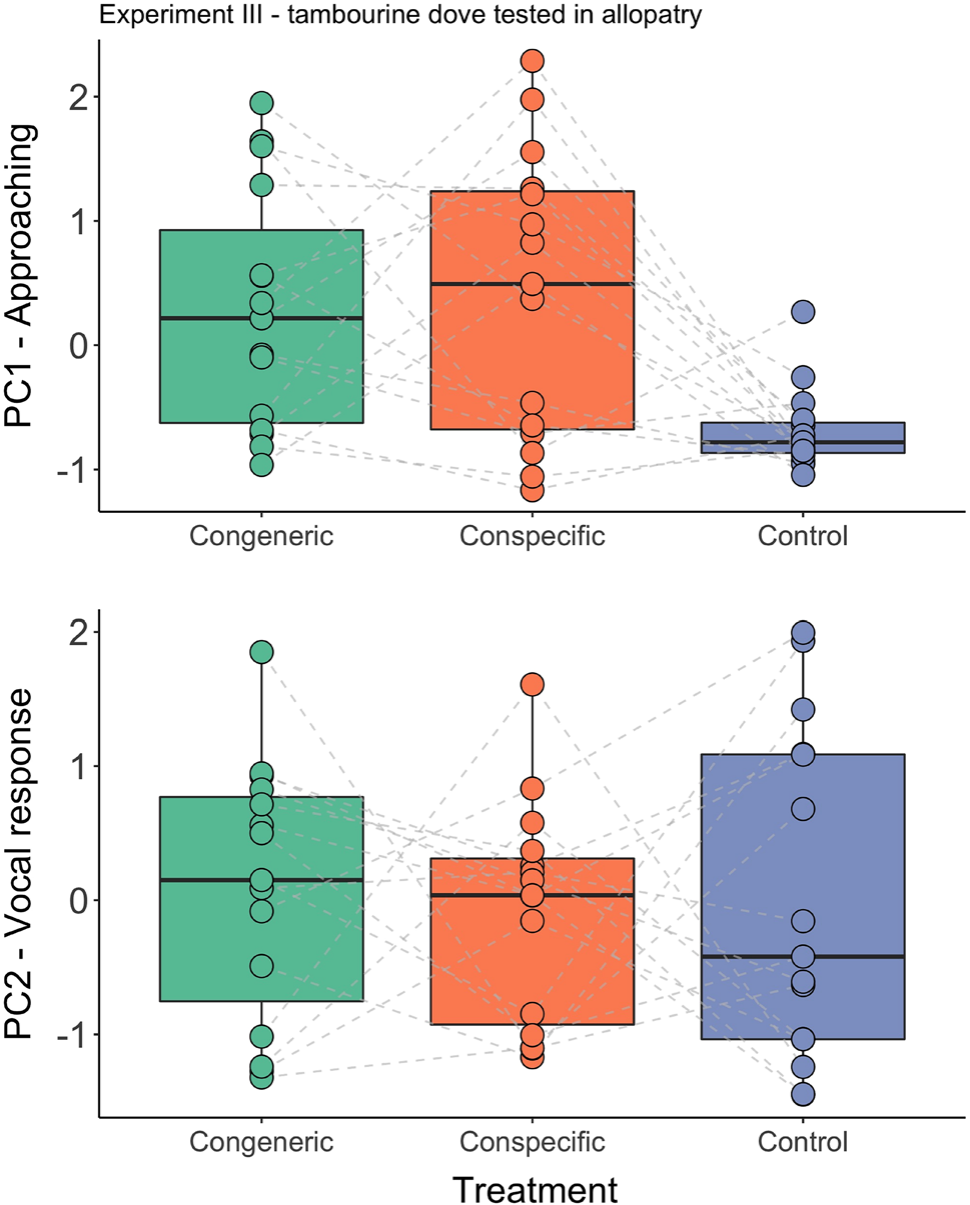
Experiment III: tambourine dove males responses to playback of conspecific, congeneric and control songs in allopatry (Uganda), measured with compound measures of response: PC1-approaching and PC2-vocal response.

The two studied species exhibited a similar approaching response (PC1) to playback of their own species in both sympatry and allopatry (all Experiments I–III; Tables 4, 5, 6). The tested birds flew close to the speaker during playback, and the closer they approached, the longer they stayed close (Figs. 2, 3, 4). In the site where the two doves are sympatric, the response pattern to the conspecific, congeneric, and control playback was very similar in both species: males approached only when conspecific songs were played, and not those of congeners or the control species (all post-hoc comparisons are presented in Table S1). In the allopatric site (no *brehmeri*), instead, *tympanistria* males demonstrated similar approach responses, and we did not find statistically significant differences in responses to playback of songs of their own species and those of the absent congener (see post-hoc tests in Table S1; Fig. 4). Furthermore, the responses to both conspecific song (*P* = 0.0002) and congeneric song (*P* = 0.0005) were significantly different from the response to the control song (details in Table S1).

**Table 4:** Experiment I: factors and interaction terms from the generalised linear mixed models used to analyse the approaching (PC1) and vocal response (PC2) of the blue-headed wood-doves living in sympatry with congener (tambourine dove) to playbacks simulating intrusion of a stranger singing conspecific, congener, and control songs.

Model	Estimate	SE	z value	Pr( >|t|)
PC1—approaching response				
(Intercept)	1.05	0.37	2.79	0.005
Treatment	− 0.56	0.17	3.33	** < 0.001**
Order	0.07	0.26	0.27	0.784
Order:treatment	0.21	0.17	1.21	0.227
PC2—vocal response				
(Intercept)	− 0.81	0.28	2.86	0.004
Treatment	0.42	0.09	4.62	** < 0.0001**
Order	− 0.11	0.09	1.24	0.214

Significant values are in bold.

**Table 5:** Experiment II: factors and interaction terms from the generalised linear mixed models used to analyse approaching (PC1) and vocal response (PC2) of the tambourine dove living in sympatry with congener (blue-headed wood-doves) to playbacks simulating intrusion of a stranger singing conspecific, congener and control songs.

Model	Estimate	SE	z value	Pr( >|t|)
PC1—approaching response				
(Intercept)	1.16	0.53	2.13	0.033
Treatment	− 0.62	0.22	2.74	**0.006**
Order	0.35	0.40	0.85	0.393
Order:treatment	− 0.32	0.20	1.52	0.128
PC2—vocal response				
(Intercept)	− 0.001	0.32	0.003	0.997
Treatment	0.17	0.16	1.03	0.302
Order	− 0.17	0.16	1.00	0.317

Significant values are in bold.

**Table 6:** Experiment III: factors and interaction terms from the generalised linear mixed models used to analyse approaching (PC1) and vocal response (PC2) of the tambourine dove living in allopatry without congener (blue-headed wood-doves) to playbacks simulating intrusion of a stranger singing conspecific, congener and control songs.

Model	Estimate	SE	z value	Pr(>|t|)
PC1—approaching response				
(Intercept)	1.13	0.37	2.92	0.003
Treatment	-0.54	0.16	3.32	** < 0.001**
Order	-0.15	0.16	0.88	0.377
PC2—vocal response				
(Intercept)	-0.05	0.26	0.20	0.841
Treatment	0.05	0.18	0.26	0.793
Order	0.15	0.18	0.79	0.430

Significant values are in bold.

The vocal responses (PC2) were less consistent among species and experiments. Males of *brehmeri* approaching the playback of their own species decreased their song rate during playback (Fig. 2, Table 4), and often continued searching for an intruder after the playback finished instead of resuming singing. Differences in vocal response to the playback of their own species compared to congeners and controls were highly significant (see Table S1). In the case of *tympanistria*, males' song behaviours were quite variable, and we found no significant differences between treatments (Tables 5, 6, S1). We found that the order of treatments had no significant influence on the response of tested birds; however, leaving the order out of certain models improved the results (Tables 4, 5, 6).

## Discussion

In this study, we experimentally tested the effect of songs of conspecific, congeneric, and neutral control individuals on the territorial response of two closely related species of African wood doves living in sympatry and allopatry. We found that males of both species always responded strongly to the conspecific stimuli, while the single species tested in allopatry (*tympanistria*) also approached the speaker when the unfamiliar song of the absent congener was played. Neither species responded to the control song, providing a useful contrast to the responses to perceived incursions by conspecific and congeneric rivals.

### Interspecific territoriality

The differences we observed between the responses to playback of conspecific song compared to congeneric song and to control song in sympatry and allopatry shed new light on the mechanisms underlying song recognition for members of the same species in addition to competition and potential interspecific territoriality in non-learning birds, represented here by African wood doves. Our results do not support the resource competition hypothesis, which posits that resources are partitioned, and defended, by dominant individuals of competing species^20^. Support for this hypothesis would be found in a strong response (with approaching as a proxy of aggression) by both species to both conspecific and congeneric song when they are found in sympatry. Instead, we found that, in sympatry, the studied *Turtur* doves only approached signals of their own species, while the response to congeneric song was similarly weak (or none) as to control song.

On the other hand, the lack of a strong response from larger *brehmeri* males to the songs of their smaller congener, together with the fact that the territories of the two species do not overlap within the sympatric range, could provide partial support for the asymmetric competition hypothesis, assuming that the larger species is dominant in interspecific territoriality^18,20,24^. As we have no information about possible hybridisation between species^34^ and differences in size and colouration between *brehmeri* and *tympanistria* are substantial^28,29^, we suspect that the reproductive interference hypothesis is unlikely to be relevant for this pair of species^20^. However, the apparent approaching response of *tympanistria* to the song of the absent *brehmeri* in allopatry does provide support for the misdirected aggression hypothesis^15,20^.

It is not unusual to find asymmetry between the response to members of one’s own species and the response to related species, or even different dialects of the same species. For example, Hamao^41^ found that sympatric, related species differ in their responses to conspecific song dialects, using as models the Japanese tit (*Parus minor*) and the varied tit (*Poecile varius*). In a later study, he also found that the responses of varied tits to heterospecific songs were much weaker in sympatric populations than in allopatric ones^42^. The difference between Hamao's studies^41,42^ and the present work is that the species he tested were not congeners but confamilial (*Parus* vs *Poecile)*, and that, unlike those two species, wood doves do not learn their songs^2^. The exact degree of phylogenetic distance between interacting species is likely not very important. As revealed by Drury et al. (2020), in the context of interspecific territoriality, song as a territorial signal acts at a deeper (even between-family) evolutionary timescale. Moreover, interspecific territoriality is known to occur even between unrelated species if they are forced by local conditions to compete for resources, as was observed for the chaffinch (*Fringilla coelebs*) and great tit (*Parus major*) on islands near Scotland^43^. However, interspecific interactions between non-learning birds are less known and require deeper consideration.

### Is song learning important for territoriality?

Song production in doves does not involve cultural transmission^44,45^. However, it is worth remembering that there are different forms of learning in acoustic communication is worth remembering. Janik and Slater^46^ distinguished between production learning and two forms of contextual learning, where individuals may produce an existing signal in a new context (usage learning) or a receiver extracts a novel meaning from a signal (comprehension learning). In both cases of contextual learning, the experience based on interactions with other individuals is crucial. So it is worth considering how potential competitors might perceive the presence or absence of a species with similar requirements and signals. Although the differences in song structure between *Turtur* spp. are small, they are consistent and allow for acoustic discrimination between species. In the case of *brehmeri* and *tympanistria*, their songs overlap in duration, number of notes, and frequency, and both species' songs can be divided into two distinct parts (own unpublished data). However, they clearly differ in the pattern of within-song note production over time (Fig. 1). In *brehmeri* songs, pauses between notes decrease constantly with time, while in *tympanistria*, pauses in the initial part of songs are variable in length. Moreover, *tympanistria* individuals differ greatly from one another in these between-note intervals, which allows for individual recognition^35^. We do not yet understand the mechanisms of species recognition in doves, particularly whether the response to conspecific songs is inherited or learned. However, pigeons are known to pay attention to the intervals between pulses, and it is likely that the above-mentioned differences in the time pattern of notes are important for species recognition^47,48^. For example, the perch-coo vocalisations of *Streptopelia* doves are long-range signals that are used for species recognition based solely on acoustic cues, and their temporal parameters were found to be the most salient features for recognition^49–51^. The song of the *Turtur* doves we studied here seems to be the functional equivalent of the perch-coo of *Streptopelia* doves, i.e. a signal that is produced to reach a receiver in the distance, who at the moment of calling is usually out of sight^52^.

Doves are known to use the same brain areas for recognition of species-specific vocalisations as songbirds^53,54^. Experiments on *Streptopelia* doves indicated that they are capable of learning discrimination tasks in an operant set-up based on conspecific and heterospecific vocalisations^49,55^. Here, the contrast we observed between *tympanistria*’s lack of response to congeneric songs in sympatry and the strong response in allopatry suggests that *tympanistria* living without related species nearby cannot differentiate between the song of their own species and the song of congeners. In the allopatric site, we observed a clear movement response to *brehmeri* playback, comparable to the response to conspecifics. Hence, our results suggest that individuals with no previous experience of interacting with the congeneric species categorised songs of similar general structure as meriting a response. At least at first, when they heard the song of the congener, they did not appear to perceive it as different from their own. Consistently, this also means that the lack of *tympanistria* response to *brehmeri* song in sympatry is a form of a learned 'not responding' by the meaning of contextual learning sensu Janik and Slater^46^.

Similar experiments on non-learning bird species are scarce. However, one interesting example was reported for flycatchers belonging to genus *Empidonax*. These members of the Tyrannidae family (and Suboscines) are known to develop species-specific songs without learning^56,57^. Initial studies on alder (*Empidonax alnorum*) and willow (*E. trailii*) flycatchers revealed little response to heterospecific song playback in sympatry^58^. However, when both species were tested in areas where they shared habitats (i.e., overlap in micro-scale), they were found to respond aggressively to heterospecific songs^59^.

Even more remarkable was the recent finding on sympatric rallids, which are also non-learners (phylogenetically even more distant from any song-learning bird taxa than Tyrannids). Jedlikowski et al. (2022) showed that the water rail, *Rallus aquaticus*, and the little crake, *Zapornia parva*, are able to distinguish not only each other but also specific individuals of the other species. This work provided an example of a dear-enemy effect in different species. Based on these studies, it seems that being a non-learner in the context of the acquisition of vocalisation (socially learned vs inherited) does not necessarily limit a bird’s abilities to diversify its responses toward different individuals of the same and even other, potentially competing, species.

The response to heterogenic song was also tested experimentally in other African dove taxa, although in a different context than in our study: hybridisation. In Uganda, de Kort et al.^52,61^ studied two closely related doves (vinaceous dove, *Streptopelia vinacea*, and ring-necked dove, *S. capicola*) as well as hybrids between the two. The authors focused on perch-coo and bow-coo signals, which both play a role in inter- and intra-sexual signalling but are produced in behaviourally different contexts (long vs short distance communication). They found that allopatric populations showed a stronger response to conspecific than to heterospecific perch-coos, but equal responses to bow-coos of either species. Instead, hybrids exhibited no clear pattern between their own coo structure and that of the species to which they responded most strongly, indicating a lack of behavioural coupling. Interestingly, in allopatry, ring-necked doves responded more strongly to perch-coos of vinaceous doves than vice versa, which the authors explained based on their ecological history. Namely, the ring-necked dove had expanded into the vinaceous dove’s range, thus creating a context in which rapid learning of a novel (heterospecific) competitor’s song might be favoured^52^.

Overall, then, research on other non-learning bird species has revealed flexibility in their response patterns to conspecific and congeneric (or even more evolutionary distant) individuals depending on the ecological and evolutionary context.

### An ecological perspective on interactions between wood doves

In the sympatric area, where *brehmeri* and *tympanistria* co-occur and their territories are often adjacent, we observed no approaching response to both congeners' songs and control songs in our experiments. From several observation posts, males of both species could be heard simultaneously, but the distance between song posts was typically more than 50–100 m, and their territories seemed not to overlap. In several patches, only one of the two species was present. These observations have two potential explanations: (i) both species can discriminate between the songs of conspecifics and those of congeners, or (ii) the weaker competitor can discriminate, and it uses this information to make decisions regarding settlement in sympatry. Our field data suggest that in Kakum NP, *brehmeri* males choose territories deeper in the forest, containing the highest trees close to streams, while *tympanistria* were often found close to forest edges or partly open areas, e.g., in tree fall gaps (or secondary forest). In Kibale NP, instead, *tympanistria* were most often found in sites like those preferred by *brehmeri* in Kakum NP. This suggests that, in sympatry, smaller *tympanistria* males may avoid settlement close to (or within the territory of) *brehmeri*, perhaps as the result of initial competitive interactions with their congeners. Such a mechanism would support the asymmetric competition hypothesis, with the caveat that the birds do not have to compete all the time. Data on the life history of wood doves are scarce but based on re-captures and recording the same birds in the same territories year after year, it appears that their territories are stable for a long time, maybe even their whole lives. Like many birds in the tropics, they are likely to be long-lived if they succeed in reaching adulthood^62^. This may suggest that the response pattern to congeners (and members of their own species) is settled when young males try to establish their territories for the first time. Based on this, and on the observations we made in our experiments, we hypothesise that when the two species are found in sympatry, young *tympanistria* likely interact with *brehmeri* males but are chased away by the stronger rival, and thus avoid such confrontations later in life. The ability to discriminate between conspecific and congeneric individuals is thus acquired through life experience, enabling the bird to save time and energy later in life. This is a hypothetical but probable scenario for the coexistence of these two species in sympatry, which also explains the pattern of responses observed in allopatry.

### Evolutionary perspective (functionality of response to congener's song in allopatry)

Our results suggest that tambourine doves may have some inherited template memory, but that response control is likely to develop while listening and interacting with potential rivals and/or mates. Therefore, we can assume that in sympatry, *tympanistria* must have learned not to respond to a song of a stronger (congeneric) rival (comprehension learning)^46^. This ability to learn responses to songs similar to their own opens the door for between-species interactions and territoriality in case of changes in species range. From this point of view, we should reconsider whether or not responding to a sister species in the allopatric zone can truly be considered maladaptive, as argued by the misdirected aggression hypothesis. In Kibale NP, where the congener is not naturally found, this response could indeed be viewed as maladaptive. Still, from a long-term and broad-scale perspective, this pattern may represent a plastic ability to find the best response in a changing environment that may or may not contain individuals of both species. Therefore, the misdirected aggression hypothesis may not be, by definition, maladaptive. Species ranges can change as a result of many factors, including in some dove species^63,64^, and the ability to adapt (i.e., to respond functionally) to a new competitor may be crucial on the evolutionary time-scale. In this context, our experimental results are consistent with a recent comparative study that revealed, through a large-scale phylogenetic analysis, that interspecific territoriality is widespread in birds and is strongly associated with hybridisation and breeding habitat overlap^18^.

The pair of *Turtur* species studied here seems to be a good model for investigating responses to heterospecific song under different forms of allopatry. *T. tympanistria* has a larger species range and less-specific habitat preferences than *brehmeri*; it can thus be found at a distance of hundreds of kilometres to the closest *brehmeri* population, as well within basically the same area but at different elevations, such as on Mount Cameroon^28,29,65^. It would be extremely valuable to understand how *tympanistria* from different allopatric populations respond to the song of congeners. For example, a study on sibling species of African sunbirds revealed that responses to heterospecific song could be strongly different among allopatric populations^66^. Conversely, another tropical species, the white-eared ground-sparrow (*Melozone leucotis*) used information encoded in vocalisations to discriminate competitor from noncompetitor species even in the absence of previous experience (understood as living in sympatry or allopatry with Prevost’s ground-sparrow, *Melozone biarcuatum*)^67^. From these previous studies and the current work, it appears that the responses of birds to heterospecific song are diverse and cannot be generalised to a single common pattern.

## Conclusions

Two species of *Turtur* wood doves living in sympatry, where they do not exhibit interspecific territoriality (at least as adults with established territories), responded strongly to songs of their own species but did not approach the song of congeners and control songs. In allopatry, instead, *tympanistria* males 'misdirected' their response to the congeneric dove song, even though they had not previously had any earlier contact with the species. Their approach response to the playback of *bremeri* song was similar to that of their own species, while they did not approach control songs. We suggest that this is not necessarily a totally maladaptive behaviour as, from an evolutionary perspective, such an ability could be useful for resolving competing interests with congeners. It is more adaptive to be plastic in the response to a potential competitor than to respond or not respond in a fixed way.

### Supplementary Information

Supplementary Figure 1.

Supplementary Table 1.

## Data Availability

The datasets analysed during the current study are available from the corresponding author on reasonable request (Tomasz S. Osiejuk, email: osiejuk@amu.edu.pl).
