# Supplementary material for: Response of forest Turtur doves to conspecific and congeneric songs in sympatry and allopatry
Source: Sci Rep. 2023 Sep 24;13:15948. doi: 10.1038/s41598-023-43035-8 (PMC10518307; doi:10.1038/s41598-023-43035-8)

Figure 1S. Spectrograms illustrating individual differences between males of the blue-headed wood-dove (*Turtur brehmeri*) and the tambourine dove (*Turtur tympanistria*). Different colours indicate the songs of the same individuals.


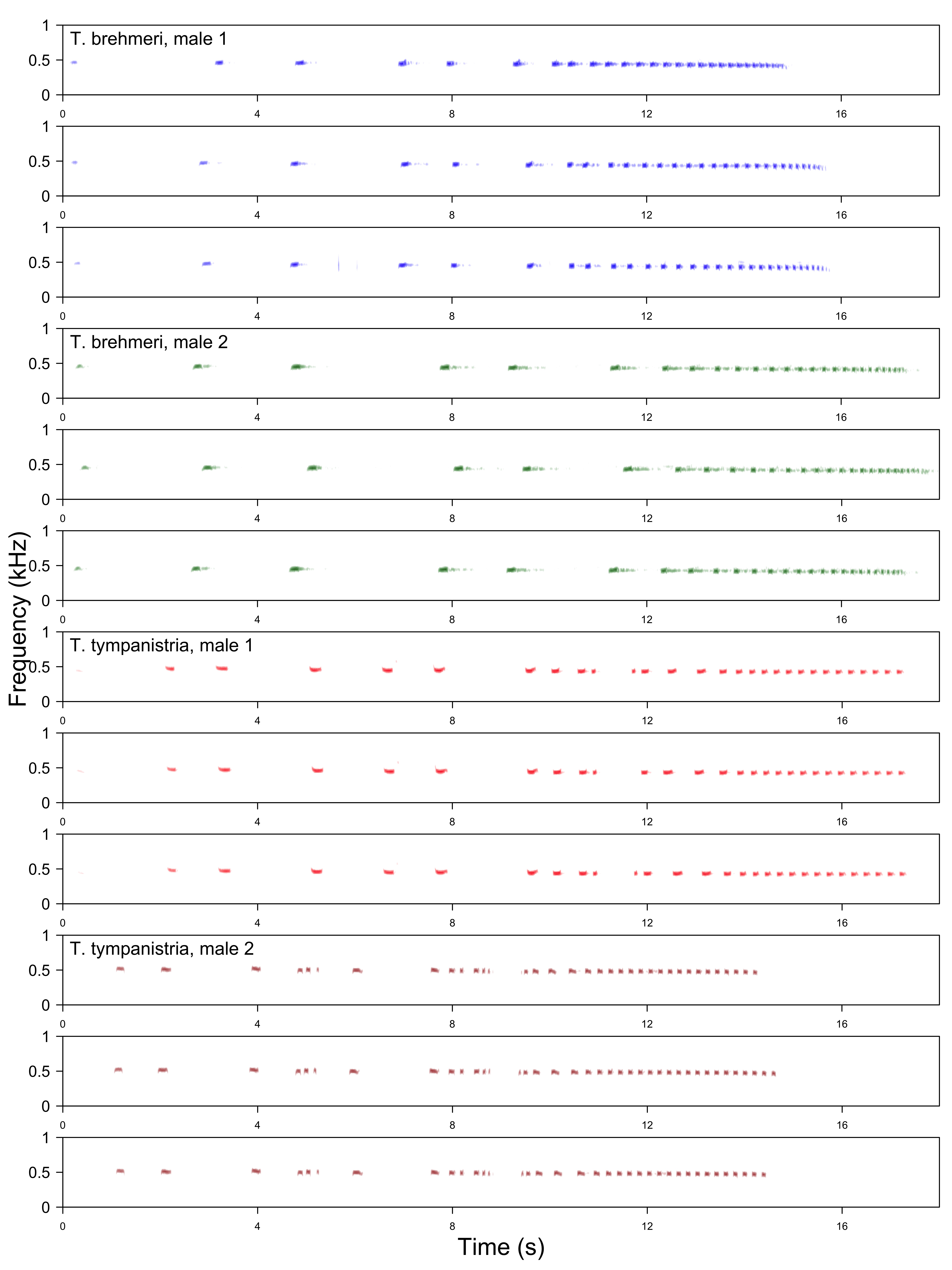

Supplement: Supplementary file 1 — Supplementary Figure 1. [file 41598_2023_43035_MOESM1_ESM.docx]
