# Supplementary material for: Response of forest Turtur doves to conspecific and congeneric songs in sympatry and allopatry
Source: Sci Rep. 2023 Sep 24;13:15948. doi: 10.1038/s41598-023-43035-8 (PMC10518307; doi:10.1038/s41598-023-43035-8)
Supplement: Supplementary file 2 — Supplementary Table 1. [file 41598_2023_43035_MOESM2_ESM.docx]

Table S1. The contrast linear comparisons of PC1 - Approaching response and PC 2 - Vocal response in Experiments I to III. Post-hoc tests were conducted to compare pairs of treatments within each experiment, i.e., Conspecific vs Congeneric, Conspecific vs Control and Congeneric vs Control.

| Experiments and post-hoc comparisons for response variables (PC1, PC2) tested | Estimate | SE | χ^2^ | *P* > χ^2^ |
| --- | --- | --- | --- | --- |
| Experiment I |  |  |  |  |
| *Model PC1 - Approaching response* | | | | |
| Conspecific vs Congeneric | 1.03 | 0.21 | 23.76 | **< 0.0001** |
| Conspecific vs Control | 1.12 | 0.21 | 28.79 | **< 0.0001** |
| Congeneric vs Control | 0.09 | 0.21 | 0.20 | 0.651 |
| *Model PC2 - Vocal response* | | | | |
| Conspecific vs Congeneric | -0.65 | 0.16 | 16.59 | **< 0.0001** |
| Conspecific vs Control | -0.83 | 0.16 | 27.17 | **< 0.0001** |
| Congeneric vs Control | -0.18 | 0.16 | 1.22 | 0.268 |
|  |  |  |  |  |
| Experiment II |  |  |  |  |
| *Model PC1 - Approaching response* | | | | |
| Conspecific vs Congeneric | 1.01 | 0.27 | 13.86 | **0.0002** |
| Conspecific vs Control | 1.36 | 0.25 | 30.12 | **< 0.0001** |
| Congeneric vs Control | 0.34 | 0.27 | 1.64 | 0.201 |
| *Model PC2 - Vocal response* | | | | |
| Conspecific vs Congeneric | -0.01 | 0.31 | 0.00 | 0.982 |
| Conspecific vs Control | -0.19 | 0.28 | 0.48 | 0.488 |
| Congeneric vs Control | -0.19 | 0.31 | 0.38 | 0.537 |
|  |  |  |  |  |
| Experiment III |  |  |  |  |
| *Model PC1 - Approaching response* | | | | |
| Conspecific vs Congeneric | 0.06 | 0.27 | 0.04 | 0.833 |
| Conspecific vs Control | 1.01 | 0.28 | 13.50 | **0.0002** |
| Congeneric vs Control | 0.96 | 0.27 | 12.17 | **0.0005** |
| *Model PC2 - Vocal response* | | | | |
| Conspecific vs Congeneric | -0.12 | 0.35 | 0.11 | 0.736 |
| Conspecific vs Control | -0.06 | 0.35 | 0.03 | 0.859 |
| Congeneric vs Control | 0.05 | 0.35 | 0.03 | 0.873 |
